# Characterization of the Gut Microbiota of Papua New Guineans Using Reverse Transcription Quantitative PCR

**DOI:** 10.1371/journal.pone.0117427

**Published:** 2015-02-06

**Authors:** Andrew R. Greenhill, Hirokazu Tsuji, Kiyohito Ogata, Kazumi Natsuhara, Ayako Morita, Kevin Soli, Jo-Ann Larkins, Kiyoshi Tadokoro, Shingo Odani, Jun Baba, Yuichi Naito, Eriko Tomitsuka, Koji Nomoto, Peter M. Siba, Paul F. Horwood, Masahiro Umezaki

**Affiliations:** 1 School of Applied and Biomedical Sciences, Federation University, Churchill, Australia; 2 Papua New Guinea Institute of Medical Research, Goroka, Papua New Guinea; 3 Yakult Central Institute, Tokyo, Japan; 4 Faculty of Nursing, The Japanese Red Cross Akita College of Nursing, Akita, Japan; 5 Department of Human Ecology, The University of Tokyo, Tokyo, Japan; 6 Faculty of Letter, Chiba University, Chiba, Japan; 7 Research Institute for Languages and Cultures of Asia and Africa, Tokyo University of Foreign Studies, Tokyo, Japan; 8 University of Tübingen, Tübingen, Germany; Catalan Institute for Water Research (ICRA), SPAIN

## Abstract

There has been considerable interest in composition of gut microbiota in recent years, leading to a better understanding of the role the gut microbiota plays in health and disease. Most studies have been limited in their geographical and socioeconomic diversity to high-income settings, and have been conducted using small sample sizes. To date, few analyses have been conducted in low-income settings, where a better understanding of the gut microbiome could lead to the greatest return in terms of health benefits. Here, we have used quantitative real-time polymerase chain reaction targeting dominant and sub-dominant groups of microorganisms associated with human gut microbiome in 115 people living a subsistence lifestyle in rural areas of Papua New Guinea. Quantification of *Clostridium coccoides* group, *C. leptum* subgroup, *C. perfringens*, *Bacteroides fragilis* group, *Bifidobacterium*, *Atopobium* cluster, *Prevotella*, Enterobacteriaceae, *Enterococcus*, *Staphylococcus*, and *Lactobacillus* spp. was conducted. Principle coordinates analysis (PCoA) revealed two dimensions with *Prevotella*, clostridia, *Atopobium*, Enterobacteriaceae, *Enterococcus* and *Staphylococcus* grouping in one dimension, while *B. fragilis*, *Bifidobacterium* and *Lactobacillus* grouping in the second dimension. Highland people had higher numbers of most groups of bacteria detected, and this is likely a key factor for the differences revealed by PCoA between highland and lowland study participants. Age and sex were not major determinants in microbial population composition. The study demonstrates a gut microbial composition with some similarities to those observed in other low-income settings where traditional diets are consumed, which have previously been suggested to favor energy extraction from a carbohydrate rich diet.

## Introduction

The human digestive system comprises a huge number of bacteria (∼10^14^) which interact closely with the host to impact on our health. The gut microbiome aids digestion, thus delivering nutrients and vitamins; and helps prevent infection through protection against colonization and modulation of the immune system [1,2]. In addition to these fundamental roles, we are now recognizing the broader implications for human health that the gut microbiome may have. Over the past 10–12 years the potential interactions between the gut microbiome and non-communicable diseases such as obesity and autism spectrum disorder have been of great interest [3–6]. These and other studies have highlighted the need for a better understanding of the core composition of the gut microbiota, and the impact of diet and other factors on gut composition. As a consequence, and as a result of rapidly advancing techniques, there has been a rapid increase in studies investigating the microbiome of the human digestive system in recent years.

Researchers have sought descriptions of the composition of the gut microbiota, resulting in the notion of enterotypes, i.e. clusters of microbes in which the composition of the population is ‘driven’ by a genus or group of specific bacteria [7]. Initially three principle human enterotypes were proposed; and were considered ‘cross-national’, in that they were not geographically confined. However, the conclusions were drawn from only 39 individuals, all from high-income countries. One year later, an independent study [8] based on 98 individuals looked at the impact of diet on gut microbial composition, and sought to determine the validity of the previously proposed enterotypes [7]. Two major findings of the study were that the gut composition was better described by two enterotypes; and these enterotypes correlated with long-term diet. The two predominant enterotypes were a *Bacteroides*-dominated type and a *Prevotella*-dominated type. The *Bacteroides*-dominated enterotype was associated with high protein and animal fat consumption, whereas individuals with a high carbohydrate intake more commonly had a *Prevotella* dominated enterotype [8].

Prior to the proposal of enterotypes, a difference between the gut microbiota of European children to that of children consuming a traditional high fiber diet in Burkina Faso was demonstrated [9], suggesting that gut microbial composition is not consistently cross-national. In the Burkina Faso children the predominant species included *Prevotella* spp. and *Xylanibacter* spp., bacteria able to hydrolyse cellulose and xylan. It was hypothesized that this *Prevotella*-rich gut flora may enable children to maximize energy intake from their carbohydrate and fiber rich diet.

Despite the role the gut microbiome plays in human health, to date most studies investigating the microbial composition have focused on people living in socio-economically developed countries. Relatively few studies have investigated the composition of the gut microbiota in low socio-economic settings where the greatest improvement in health outcomes is needed; and specifically, where the burden of infectious diseases remains high. Based on the limited data to date, it appears that there are differences in the composition of gut microbiota, both within high-income European countries [10]; and particularly when comparing people from socio-economically developed settings to those that live a non-Western or traditional subsistence lifestyle [9,11]. Moreover, when detailed analyses have been conducted in low socio-economic settings, typically studies have focused on children and sample sizes have been small [9,12,13]. Two recent studies in Malawi have used appreciable sample sizes, although with the focus on children only [14,15]; with only one sizable study looking at adults and children from high- and low-income settings [16]. Thus, there remain large gaps in our knowledge in this field, particularly from the Asia-Pacific region. To address these issues, we have used reverse transcriptase real-time PCR to determine the prevalence of the recognized important groups of bacteria in the gut of adults and children in the highlands and lowlands of Papua New Guineans living a predominantly traditional lifestyle.

## Methods

### Ethics Statement

Participation in the study was voluntary, and all participants or parent/guardian (for participants under the age of 18 years) provided written informed consent. Ethics approval was granted by the Papua New Guinea (PNG) Institute of Medical Research (IMR) institutional review board (Ethics 10.25), and the PNG Medical Research Advisory Council (Ethics # 11.25).

### Sample Collection

Samples were collected from two PNG highland regions (Asaro Valley, approximately 20 km from the provincial capital of Goroka in Eastern Highlands Province and the Tari Basin, Hela Province) and one lowland region (East Maprik, East Sepik Province). Participants from the highland regions consume sweet potatoes as their staple food, supplemented by edible leafy vegetables, while those from the lowland consume sago starch and edible leafy vegetables, and various starchy crops (i.e., banana, yam, taro). The participants from both regions occasionally consume pig meat, but the contribution to total protein intake is limited [17,18].

Samples were collected in highland regions in February to March, 2012; and in the lowland region in September, 2012. Demographic information (i.e. sex, age) and history of antibiotic use was collected by face-to-face interview. Age was estimated by referring to the demographic database completed by members of the research team during earlier fieldwork [17,19]. Height was measured to the nearest 1 mm using a field anthropometer (GPM, Switzerland). Weight measurements were taken to the nearest 0.1 kg using a digital scale (Tanita Japan), without shoes and in light clothing. Body Mass Index (BMI) was calculated as a function of body weight (kg) divided by squared height (m) and rounded to 1 decimal place.

Participants were given a clean plastic bag for faecal collection at the participant’s earliest convenience. Once a stool specimen had been passed it was given to a member of the study team, who preserved it in RNAlater (Life Technologies) to stabilise nucleic acids. Initially a 10-fold dilution (w:v) of stool was prepared in RNAlater.

### RNA extraction

A further 5-fold dilution of the stool sample in RNAlater (final ratio 1:50) was conducted. A 200 μl aliquot was washed in phosphate buffered saline, centrifuged, and the pellet stored at IMR laboratories at-80°C. The pellet was then sent to Japan for analysis. RNA was extracted using a modified method described by Matsuda et al. [20]. Briefly, the thawed sample was resuspended in a solution containing 346.5 μl of RLT buffer (Qiagen GmbH, Hilden, Germany), 3.5 μl of β-mercaptoethanol, and 100 μl of Tris-EDTA buffer. Glass beads (300 mg; diameter, 0.1 mm) (Tomy Seiko, Tokyo, Japan) were added to the suspension, and the mixture was disrupted vigorously for 5min using a ShakeMaster Auto (Biomedical Science, Tokyo, Japan). 500 μl of acid phenol was added and mixed, and the mixture was incubated for 10 min at 60°C. After incubation, the mixture was added to 100 μl of chloroform-isoamilalcohol (24:1) and mixed by vortex. Following the centrifugation at 12,000 × *g* for 10 min at 4°C, 450 μl of the supernatant was collected and added to an equal volume of chloroform-isoamilalcohol. After mixing by vortex, the mixture was centrifuged at 12,000 × *g* for 5 min, 400 μl of supernatant was collected and subjected to isopropanol precipitation. Finally, the nucleic acid fraction was suspended in 200 μl of nuclease-free water (Ambion, Inc., Austin, TX, USA) and frozen at-80°C until use.

### Bacterial microbiota composition

We conducted Yakult Intestinal Flora-SCAN (YIF-SCAN) analysis based on reverse transcription-quantitative PCR (RT-qPCR) analysis using methods previously described [20,21]. In brief, primers targeting either the 16S rRNA or 23S rRNA region of the genome were used to detect dominant and sub-dominant gut microbes. Target organisms and their lower limit of detection are listed in Table 1.RT-qPCR was conducted by using an OneStep RT-PCR kit (Qiagen GmbH, Hilden, Germany). RT-PCR amplification and detection were performed in 384-well optical plates on an ABI PRISM 7900HT Sequence Detection System (Applied Biosystems, Foster City, CA, USA). The total bacterial count obtained by RT-qPCR is shown as the sum of the counts of 10 bacterial groups and one species. The count of *Lactobacillus* obtained by RT-qPCR is expressed as the sum of the counts of six subgroups and two species.

**Table 1:** Detection of bacteria using RT-qPCR.

Organism	Detection limit (log_10_/g)	Primers	Mean ± SD (log_10_/g)	Prevalence
Phylum Firmicutes				
*Clostridium coccoides* group	5.0	Matsuki et al, 2004	8.9 ± 0.7	98%
*C*. *leptum* subgroup	5.0	Matsuki et al, 2004	9.3 ± 0.7	99%
C. perfringens	2.6	Matsuda et al, 2009; Kikuchi et al, 2002	4.5 ± 1.2	66%
*Enterococcus* spp	3.0	Matsuda et al, 2009	6.5 ± 1.5	84%
*Staphylococcus* spp	3.0	Matsuda et al, 2009	4.2 ± 0.8	53%
*Lactobacillus gasseri* subgroup	2.3	Matsuda et al, 2009	4.1 ± 1.1	54%
*L*. *reuteri* subgroup	2.7	Matsuda et al, 2009	4.7 ± 1.3	51%
*L*. *ruminis* subgroup	2.3	Matsuda et al, 2009	6.0 ± 1.6	66%
*L*. *plantarum* subgroup	2.3	Matsuda et al, 2009	3.3 ± 1.1	3%
*L*. *sakei* subgroup	2.3	Matsuda et al, 2009	2.8 ± 0.5	8%
*L*. *casei* subgroup	2.3	Matsuda et al, 2009	3.1	1%
L. brevis	2.8	Matsuda et al, 2009	3.8 ± 1.0	3%
L. fermentum	4.0	Watanabe, 1998	4.9 ± 0.5	3%
Phylum Bacteroidetes				
*Bacteroides fragilis* group	5.0	Matsuki, 2007	6.8 ± 0.9	88%
*Prevotella* spp	4.0	Matsuki et al, 2004	9.0 ± 0.9	92%
Phylum Actinobacteria				
*Bifidobacterium* spp.	5.1	Matsuki et al, 2004	7.0 ± 1.3	70%
*Atopobium* cluster	5.9	Matsuki et al, 2004	8.1 ± 0.8	100%
Phylum Proteobacteria				
Enterobacteriaceae	4.3	Matsuda et al, 2007	7.4 ± 1.1	97%

Phylogenetic groups of organisms detected using RT-qPCR, the source of primers that were used and the limit of detection for each group. The mean number of organisms detected (± standard deviation) in faecal samples from Papua New Guinean study participants (n = 115), and the prevalence of detection is also provided.

Quantification of bacterial populations was conducted by correlating RT-qPCR outputs to previously obtained qPCR, FISH and/or culture results [20].

### Data analysis

Data were entered into a spreadsheet (Microsoft Excel) as log bacterial counts, and basic statistical analysis conducted. All statistical analysis was conducted using SPSS v20. Non Linear Principle Components Analysis, also known as Principle coordinates analysis (PCoA), was conducted using the original data and the CATPCA program in SPSS. All variables were transformed as spline ordinal (degree = 2, the number of internal knots = 2) and two components were extracted. Variables were removed from analysis if the total variance explained for the variable in the analysis was less than 25%. Relationships between gut microbial composition and age, sex, regions were sought. For age, study participants were grouped as <5 years old (children), 5–17 years old (older children and adolescents, but referred to as ‘adolescents’ to avoid confusion with the ‘children’ group), and ≥18 years (adults). Participants were categorised as either living in the highlands or the lowlands for regional analysis. Comparisons were conducted using the Mann-Whitney U test and Kruskal-Wallis test.

## Results

A total of 126 samples were collected: 29 from Asaro Valley, 60 from Tari Basin (collectively the highland samples) and 37 from Maprik (lowland samples). Of the 126 samples tested using reverse transcriptase qPCR, 11 samples failed to amplify and were excluded from analysis. An overview of the basic demographic data for the remaining 115 participants is provided in Table 2. The age range of participants was 2 to 66 years, with an average of 30 years (median 28 years). Twenty-six participants were under 18 years of age, but only three of the juveniles were under 5 years of age. The majority of participants (71/115) were male. Participants < 18 years old were excluded from BMI analysis.

**Table 2:** An overview of the demographic data of study participants, including age, sex, location and average BMI.

Location	Average age (age range) years	Sex (M;F)	BMI all adults	BMI male adults	BMI female adults
Overall	30 (2–66)	71; 44	23.27	23.16	23.45
(n = 115)			(n = 89)	(n = 54)	(n = 35)
Goroka	29 (4–66)	13; 14	23.84	23.50	24.09
(n = 27)			(n = 19)	(n = 8)	(n = 11)
Lebani)	26 (15–49)	20; 2	23.11	23.11	NA
(n = 22			(n = 14)	(n = 14)	(n = 0)
Maprik	35 (12–58)	18; 11	22.07	22.54	21.27
(n = 29)			(n = 27)	(n = 17)	(n = 10)
Tari	30 (2–65)	20; 17	24.10	23.72	24.50
(n = 37)			(n = 29)	(n = 15)	(n = 14)

BMI was calculated on adult study participants only.

Table 1 provides the average number of each bacterial group detected in the 115 study participants, and the frequency of detection (prevalence). The most abundant bacteria were the *Clostridium leptum* subgroup (log_10_ 9.3±0.7), followed by *Prevotella* spp (log_10_ 9.0±0.9), *C*. *coccoides* group (log_10_ 8.9±0.7) and *Atopobium* cluster (log_10_ 8.1±0.8). Each of these four groups of bacteria was present in ≥92% of samples. S1 Table provides the raw data.

**Fig 1:**
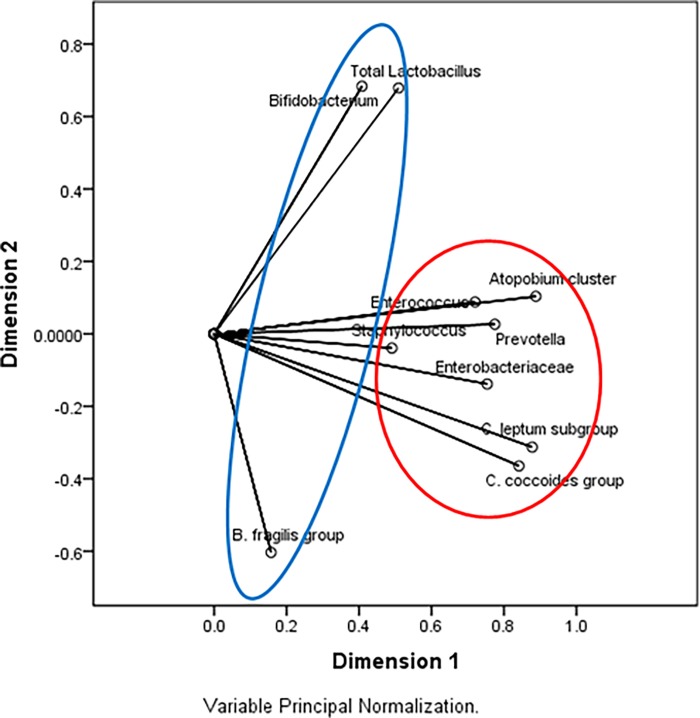
Output of PCoA showing 2 dimensions. *Prevotella* groups into dimension 1 (red circle) with other commonly detected bacterial groups such as the clostridia, Enterobacteriaceae and *Atopobium* cluster. *Bacteroides fragilis* group is a component of dimesion 2 (blue eclipse) and has an inverse relationship with *Lactobacillus* and *Bifidobacterium*.

The average number of *Prevotella* spp. was significantly greater than average number of *Bacteroides fragilis* group (log_10_ 6.8±0.9) (S1 Fig.). Individually, 101 of 115 participants had a higher number of *Prevotella* spp. than *B*. *fragilis* group. In nine of the 14 participants for which *B*. *fragilis* group was more abundant than *Prevotella* spp, the later was not detected (detection limit of log_10_5.0); and in one sample numbers of *Prevotella* spp. and *B*. *fragilis* group were comparable (log_10_8.0). Due to nonlinear relationships present between variables in the data set, PCoA was used to extract two components. *Prevotella* grouped with six other groups of bacteria tested for in this study, whereas *B*. *fragilis* grouped with two other groups (Table 3). The two components explain 62.09% of the variance in the data set. The outcome of PCoA is illustrated in Fig. 1. The results of the PCoA support an inverse relationship between *Prevotella* spp. and *B*. *fragilis* group with the two factor loading for these species having opposite signs.

**Table 3:** Principle coordinates analysis loadings for microbial groups in the gut of Papua New Guinean study participants.

	Dimension	
	1	2
*C*. *coccoides* group	0.841	
*C*. *leptum* subgroup	0.878	
B. fragilis		-0.604
Bifidobacterium		0.683
*Atopobium* cluster	0.888	
Prevotella	0.775	
Enterobacteriaceae	0.754	
Enterococcus	0.720	
Staphylococcus	0.491	
C. perfringens		
Total *Lactobacillus*		0.679

All variables considered to be spline ordinal (degree = 2, no. of internal knots = 2); VAF = 62.09%.

**Fig 2:**
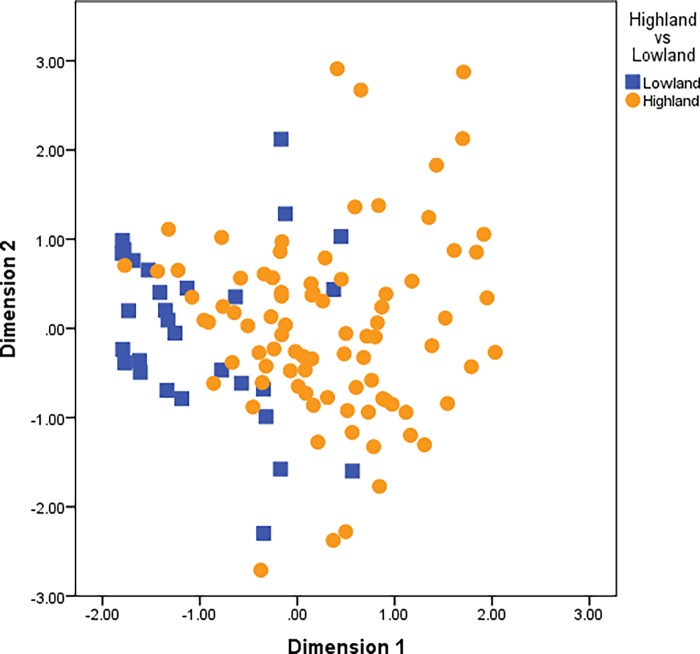
Outcomes of PCoA by region (highland vs lowland). There is a lower number of dimension 1 organisms in lowlanders than highlanders.

**Fig 3:**
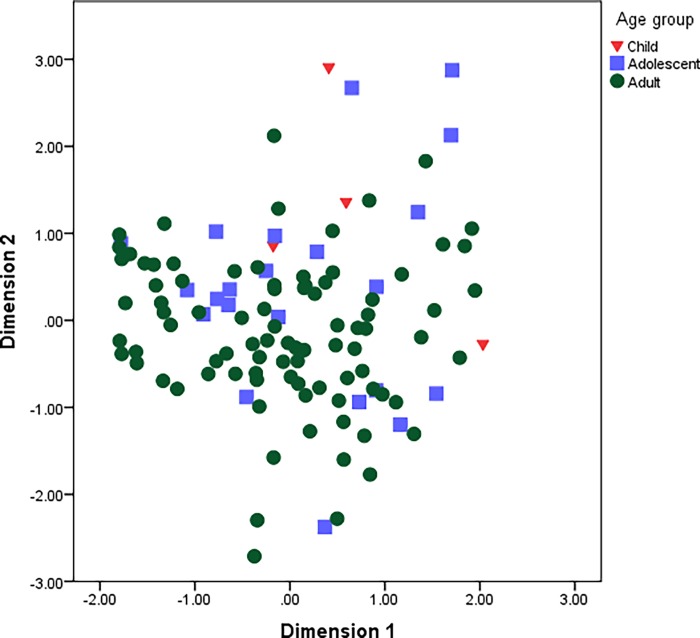
Outcomes of PCoA by age (children, adolescents and adults).

**Fig 4:**
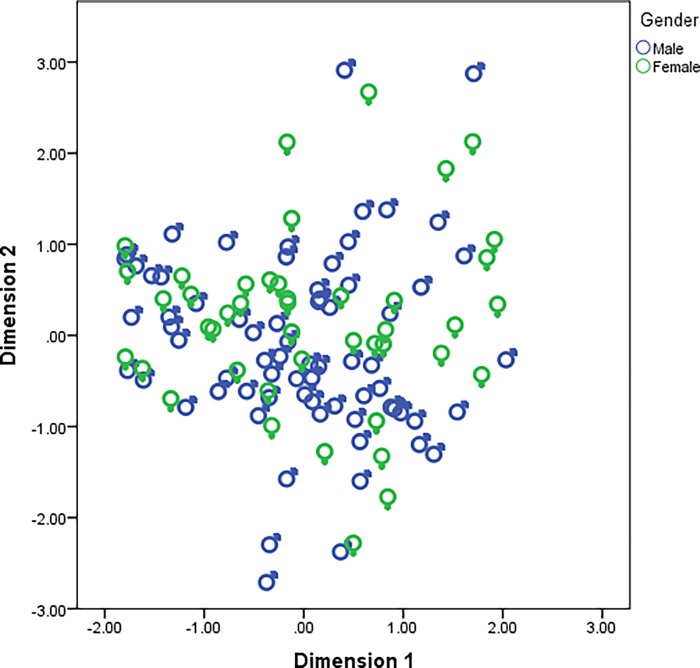
Outcomes of PCoA by sex (males and females).

Statistical analysis was conducted to determine whether socio-demographic factors (sex, region, and age) impacted on the composition of the gut microbiota. Using PCoA outcomes, clustering occurred according to region (Fig. 2). No clear trends were observable by sex or age (Figs. 3 and 4). Specific comparisons were conducted for key microbial groupings primarily based of phyla (Bacteroidetes, Firmicutes, Proteobacteria and Actinobacteria), but also using total bacterial numbers and total *Lactobacillus*. Kolmogovov-Smirnov analysis revealed non-normal distribution of data. Appropriate descriptive statistic analyses were conducted, revealing differences in multiple bacterial populations in highland and lowland populations (Table 4), and relatively fewer differences due to age (Table 5) and sex (Table 6). The differences observed in bacterial populations of highland compared to lowland individuals were also observed when only adults were included in analysis (S2 Table).

**Table 4:** Comparison of population numbers of selected microbial groups in the highland and lowland study participants.

Microbial Group	Bacteria	Bacteria	Statistical analysis	P-value
	log_10_ ± std dev	log_10_ ± std dev		
	Lowland (n = 29)	Highland (n = 86)		
Bacteroidetes	8.634 ± 0.740	9.013 ± 1.013	Mann-Whitney U test	0.005
Firmicutes	8.928 ± 0.629	9.631 ± 0.577	Mann-Whitney U test	0.000
Enterobacteriaceae	5.917 ± 2.376	7.606 ± 0.953	Mann-Whitney U test	0.000
Actinobacteria	7.461 ± 0.854	8.491 ± 0.586	Mann-Whitney U test	0.000
Total *Lactobacillus*	3.652 ± 2.588	5.443 ± 2.175	Mann-Whitney U test	0.001
Total bacteria	9.235 ± 0.621	9.866 ± 0.536	Mann-Whitney U test	0.000

**Table 5:** Comparison of population numbers of selected microbial groups in children, adolescents and adults.

Microbial Group	Bacteria	Bacteria	Bacteria	Statistical analysis	P-value
	log_10_ ± std dev	log_10_ ± std dev	log_10_ ± std dev		
	Child (n = 4)	Adolescent (n = 22)	Adult (n = 89)		
Bacteroidetes	9.15 ± 0.76	8.97 ± 0.85	8.89 ± 1.00	Kruskal-Wallis	0.936
Firmicutes	9.69 ± 0.74	9.48 ± 0.66	9.44 ±0.67	Kruskal-Wallis	0.986
Enterobacteriaceae	7.20 ± 0.99	7.36 ±1.80	7.14 ± 1.60	Kruskal-Wallis	0.469
Actinobacteria	9.16 ± 0.37	8.35 ± 0.69	8.16 ± 0.81	Kruskal-Wallis	0.030
Total *Lactobacillus*	7.53 ± 1.02	5.38 ± 2.49	4.78 ± 2.37	Kruskal-Wallis	0.033
Total bacteria	10.00 ± 0.56	9.73 ± 0.61	9.69 ± 0.63	Kruskal-Wallis	0.829

Children <5 years old; adolescents 5–17 years old; adults ≥18 years old. All Kruskal-Wallis tests were conducted on child, adolescent and adult groups.

**Table 6:** Comparison of population numbers of selected microbial groups in male and female study participants.

Microbial Group	Bacteria	Bacteria	Statistical analysis	P-value
	log_10_ ± std dev	log_10_ ± std dev		
	Male (n = 71)	Female (n = 44)		
Bacteroidetes	8.903 ± 0.880	8.941 ± 1.094	Mann-Whitney U test	0.687
Firmicutes	9.505 ± 0.638	9.371 ± 0.701	Mann-Whitney U test	0.331
Enterobacteriaceae	6.937 ± 1.766	7.573 ± 1.255	Mann-Whitney U test	0.059
Actinobacteria	8.190 ± 0.808	8.298 ± 0.787	Mann-Whitney U test	0.521
Total *Lactobacillus*	4.885 ± 2.331	5.164 ± 2.537	Mann-Whitney U test	0.461
Total bacteria	9.709 ± 0.588	9.705 ± 0.677	Mann-Whitney U test	0.991

## Discussion

To date, human gut composition studies have lacked global representation; and emphasis has been placed on very detailed analysis of often undersized sample sizes. We have applied RT-qPCR to detect and quantify dominant and sub-dominant groups of microbes known to inhabit the human gut of Papua New Guinean people living a mostly traditional subsistence-based lifestyle. Our study revealed differences in the gut composition of highland and lowland people in PNG, and differences in children and adults. Also of interest is that *Prevotella* was detected in higher numbers than *Bacteroides* in the majority (88%) of study participants, and there was an inverse relationship between the two genera.

Differences in gut microbial composition based on location and age of study participants have been observed in other studies, though geographical variation has more commonly been investigated at the inter-country level rather than the intra-country level [9,16]. In this study we compared populations with similar socio-economic conditions and subsistence lifestyle. Of the seven phylogenetic groups that cluster in dimension 1 (Table 3), five were present in the vast majority of samples analysed (≥92%), and the enterococci were present in 84% of samples (Table 1). This component represents the organisms that are commonly present, and often present in high numbers relative to many of the other organisms detected in this study. The staphylococci were an exception to this observation; they clustered in dimension 1 despite a relatively low rate of detection and mean bacterial numbers. Organisms in dimension 1 (excluding staphylococci) contributed to the core gut microbiota of people in PNG.

In Papua New Guineans *Prevotella* predominated over *Bacteroides*: this is likely a reflection of the diet and subsistence lifestyle of the study participants. De Filippo et al [9] found *Prevotella* (along with *Xylanibacter* and *Treponema*) to be present in children from Burkina Faso but not in European children. The authors hypothesized that the presence of these genera were on account of the high fiber diet, and that the bacteria maximize energy extraction from plant polysaccharides. Wu and colleagues [8] recently confirmed a link between *Bacteroides*-rich enterotypes and high protein, high animal-fat diets, and *Prevotella*-rich enterotypes with high carbohydrate diets. Although “western foods” such as rice and tinned meat are available and commonly consumed in PNG, these foods contribute more to the diet of urbanized Papua New Guineans than people living a rural subsistence lifestyle. For the latter, the focus of this study, garden foods continue to primarily contribute to daily energy intake [21,22]. As such, much of the diet consists of complex carbohydrates rather than refined carbohydrates; and protein intake is lower than in western diets [23]. A food frequency questionnaire recently conducted by our research team in the highland study sites confirmed that people living it these communities do not meet the biologically required protein intake [24].

Bäckhed et al [25] observed that *Prevotella* and *Bacteroides* coexist if the community is predominant in Firmicutes, whereas in communities dominated by Bacteroidetes the two Gram negative genera are mutually exclusive. In our study participants *Prevotella* and *Bacteroides* coexisted (both organisms present in 80% of samples), and Firmicutes were detected in higher numbers than Bacteroidetes. However, our methods did not comprehensively detect all bacteria within the Firmicutes and Bacteroidetes, thus it is difficult to make a direct comparison to other settings.

Although always represented in PNG samples and typically in high numbers, there are lower numbers of bacteria clustering in dimension 1 in lowland participants than in highland participants (Fig. 2 and Table 4). The causes of the difference between highland and lowland populations are difficult to ascertain in this study. People living in the PNG highlands (1,500–2,300 meters altitude) have a diet that differs to some degree from the lowland diet, though both are subsistence based and are broadly similar (high in carbohydrates, the primary source of carbohydrate varies; and low in protein). The homology/heterogeneity of the core gut microbiome across the genetic spectrum has not been fully elucidated [7,9]. If genetic factors influence human microbiome composition this may be a contributing factor; as PNG and nearby island communities are renowned for the genetic diversity of their people. In general lowland people have recent Austronesian influences on their genetic composition (including non-Papuan Melanesian, Polynesian and Micronesian influences), relative to the more remote highland people in whom genetic composition is more homogenous [26–28]. The true extent of microbial composition differences between highland and lowland populations, and the mechanisms of variation, warrants further investigation, ideally in conjunction with detailed analysis of dietary intake.

We observed differences in Actinobacteria (*Bifidobacterium* and *Atopobium*) and *Lactobacillus* in children, adolescents (children over 5 to 17 years old) and adults (Table 5). These findings correlate with recent findings, in which changes in the composition of the gut flora are shown to occur with age. Perhaps the greatest change occurs at the time of weaning, prior to which *Bifidobacterium* (or other genera of Actinobacteria) constitute a major proportion of the gut flora [9]. However, the change is not as abrupt as once thought, with *Bifidobacterium* being more abundant in adolescents than adults in a study conducted in the USA [29]. Differences in gut composition have also been noted in later life, though in participants of a greater age than our most senior study participants [30].

Our lactobacilli primer set detects a broad range of *Lactobacillus* spp. [20], included those identified as major genera in the human gut in independent studies [31,32]. Thus we were able to gain an approximation of the overall number of lactobacilli while also determining the predominant subgroups. In doing so we have shown *L*. *ruminis* subgroup to be the most prevalent and abundant group of lactobacilli in our study participants relative to other groups.

The association with increased Firmicutes in obese humans and animal models [4,6] has led to speculation about a role for these organisms in obesity. The majority of studies have been conducted in high income settings; however, Xu and colleagues [33] used quantitative PCR to demonstrate reduced Bacteriodetes and an increased Firmicutes:Bacteroidetes ratio in obese school children in the minority Kazakh people from under-developed farming communities in rural China. Our analyses did not use universal primers to target all Firmicutes and Bacteroidetes, thus it is difficult to interpret how our findings pertain to obesity. Moreover, the relationship between Firmicutes:Bacteroidetes ratio and obesity is more complex than initially thought [34,35]. Nonetheless, it is possible that high numbers of Firmicutes may be an evolutionary advantage in people living a truly subsistence lifestyle (such as the people of PNG) due to an increased energy harvesting efficiency.Metagenomic approaches are required to further elucidate relationships between gut microbiota and obesity in this and other Pacific populations, where obesity and type 2 diabetes are becoming problematic.

The application of RT-qPCR does not enable the detection of all bacteria in the gut, only those targeted by the primers used (Table 1). The primers used in this study have been shown to detect a large range of bacteria, albeit culturable species. However, the primers have been shown to amplify various species within the target taxonomic group, while being specific for the target group [36,37]. As such, the primers used in our study are likely to amplify closely-related non-culturable species should they be present. Importantly, the higher taxonomic groups found to predominate in this study using RT-qPCR are consistent with the findings of other studies using sequence based technologies and microarrays [8,38,39]; and Tap et al [40] found cloning-based 16S rRNA sequence data to closely match qPCR data for the dominant species in their study. Thus, while this analysis does not allow for the detection of the full breadth of microbes present, we have been able to characterize the gut microbiota based on the predominant groups.

Our study documents important observations regarding the composition of the gut microbiota of people living a subsistence lifestyle in PNG, in particular the predominance of *Prevotella* over *Bacteroides* and an insight into the core gut microbiota. Future studies are warranted using PCR independent detection methods that accurately characterize the entire gut microbiome as opposed to targeting selected dominant and sub-dominant microbial taxa; and ideally correlate gut microbiome composition to diet and activity. Recent studies have begun exploring the interactions of gut microbiota with nutritional status and infectious diseases in Malawi [15,22]: further work should address such issues in other low-income settings such as PNG where the burden of infectious disease is high.

## Supporting Information

S1 Fig Box plot comparing the average number (log_10_/g faeces) of *Bacteroides* compared to *Prevotella* in study participants (n = 115). (TIF)

S1 Table Quantification of bacterial populations using reverse transcriptase real time PCR for all individuals included in analysis. (DOCX)

S2 Table Comparison of population numbers of selected microbial groups in adult highland and lowland study participants. Children and adolescents excluded from analysis (n = 89).
(XLSX)
