# Supplementary figures and images for: Characterization of the Gut Microbiota of Papua New Guineans Using Reverse Transcription Quantitative PCR
Source: PLoS One. 2015 Feb 6;10(2):e0117427. doi: 10.1371/journal.pone.0117427 (PMC4319852; doi:10.1371/journal.pone.0117427)

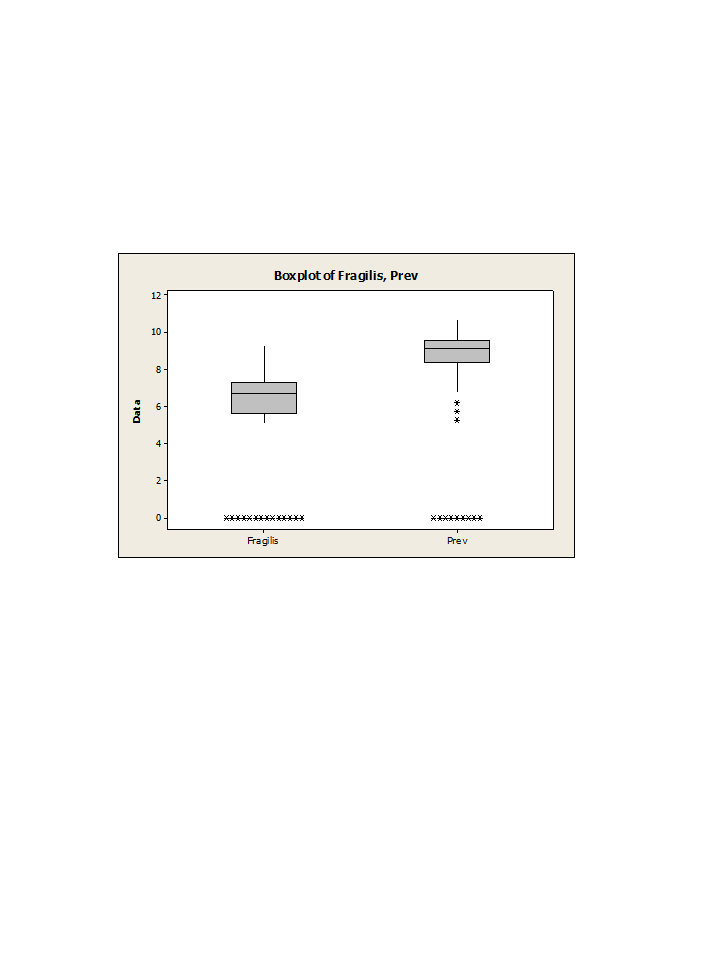

Supplement: S1 Fig — (TIF) [file pone.0117427.s001.tif]
