# Supplementary material for: Characterization of the Gut Microbiota of Papua New Guineans Using Reverse Transcription Quantitative PCR
Source: PLoS One. 2015 Feb 6;10(2):e0117427. doi: 10.1371/journal.pone.0117427 (PMC4319852; doi:10.1371/journal.pone.0117427)
Supplement: S1 Table — (DOCX) [file pone.0117427.s002.docx]

| Microbial Group | Bacteria  log_10_ ± std dev | Bacteria  log_10_ ± std dev | Statistical analysis | P-value |
| --- | --- | --- | --- | --- |
|  | Lowland (n=29) | Highland (n=86) |  |  |
| Bacteroidetes | 8.634 ± 0.740 | 9.013 ± 1.013 | Mann-Whitney U test | 0.007 |
| Firmicutes | 8.928 ± 0.629 | 9.631 ± 0.577 | Mann-Whitney U test | 0.000 |
| Enterobacteriaceae | 5.917 ± 2.376 | 7.606 ± 0.953 | Mann-Whitney U test | 0.000 |
| Actinobacteria | 7.461 ± 0.854 | 8.491 ± 0.586 | Mann-Whitney U test | 0.000 |
| Total *Lactobacillus* | 3.652 ± 2.588 | 5.443 ± 2.175 | Mann-Whitney U test | 0.003 |
| Total bacteria | 9.235 ± 0.621 | 9.866 ± 0.536 | Mann-Whitney U test | 0.000 |
